# The relationship between gluten-free diet and IgA nephropathy: A review

**DOI:** 10.1097/MD.0000000000041964

**Published:** 2025-06-13

**Authors:** Tianqi Pei, Dengzhou Guo

**Affiliations:** a Graduate School, Hebei University of Chinese Medicine, Shijiazhuang, China; b Hebei Provincial Hospital of Traditional Chinese Medicine, Shijiazhuang, China; c Hebei Technology Innovation Center of TCM Spleen and Kidney Diseases, Shijiazhuang, China.

**Keywords:** causal inference, gluten-free diet, IgA nephropathy, Mendelian randomization

## Abstract

The aim is to explore the causal relationship between gluten-free diet and IgA nephropathy by conducting Mendelian randomization (MR) analysis and briefly summarizing the current research on gluten-free diet and IgA nephropathy to provide new insights for the prevention and treatment of IgA nephropathy. The 64,949 samples of gluten-free diet and 477,784 samples of IgA nephropathy were obtained from the genome-wide association study. Four genetic variants strongly associated with a gluten-free diet were extracted as instrumental variables. Two-sample MR analysis was performed by multiplicative random-effects inverse variance weighted, MR-Egger regression, weighted median, weighted mode, simple median, maximum likelihood ratio, and penalized weighted median as analysis models, respectively. Odds ratio values were used to evaluate the causal relationship between gluten-free diet and IgA nephropathy. The sensitivity analysis was carried out by the Cochran *Q* test, MR-Pleiotropy RESidual Sum and Outlier test, MR-Egger intercept test, and leave-one-out analysis to assess the robustness of the results. Multiplicative random-effects inverse variance weighted analysis showed a significant negative correlation between gluten-free diet and IgA nephropathy (odds ratio = 2.333 × 10^−4^, 95% confidence interval = 4.628 × 10^−6^~1.176 × 10^−2^, *P *= 2.899 × 10^−5^). In conclusion, there might be a negative causal relationship between gluten-free diet and IgA nephropathy, which could potentially reduce the risk of IgA nephropathy.

## 1. Introduction

IgA nephropathy (IgAN), an immune-mediated glomerular disease, is the most common primary glomerulonephritis worldwide^[1]^ and the leading cause of chronic kidney disease and end-stage renal disease (ESRD).^[2]^ It is pathologically characterized by predominant IgA or IgA deposition in the mesangial area (common deposition of IgG, IgM, or complement C3) with mesangial hyperplasia. Clinical manifestations range from asymptomatic hematuria to gross hematuria with or without proteinuria, nephrotic syndrome, acute kidney injury, or rapidly progressive glomerulonephritis.^[3]^ The global incidence reaches 2.5/100,000,^[4]^ and 20% to 40% of patients develop ESRD within 20 years of diagnosis.^[5]^ IgAN has an insidious onset and is most common in young people. According to the European Renal Association Registry, IgAN accounts for the highest proportion of ESRD patients who start renal replacement therapy for primary glomerular disease.^[6]^ The prevalence of IgAN in China is about 50% of primary glomerular diseases.^[3]^ IgAN has been discovered by Berger since 1968,^[7]^ and there is still a paucity of specific treatment options in clinical practice.

Gluten is a protein complex mainly found in wheat grains, mainly gliadin and glutenin,^[8]^ and the global incidence of gluten-related disorders is approximately 5%.^[9]^ Gluten-free diet (GFD) is a dietary therapy established for the treatment of gluten-induced immune-mediated diseases such as celiac disease (CD), dermatitis herpetiformis, wheat allergy, gluten ataxia, and nonceliac gluten sensitivity disease caused by gluten proteins.^[10]^

In the late 1970s, wheat protein antibodies were first identified in the serum of a patient with IgAN and CD, and after undertaking a GFD, immune complexes in the serum decreased, revealing the source of IgAN dietary antigens,^[11]^ and the relationship between GFD and IgAN has received widespread attention. In related animal experiments and clinical trials, a multitude of studies have suggested that dietary antigens (e.g., gliadin) can induce the development of IgAN, and GFD can reduce IgA immune complexes (IgA ICs) in the mesangium and circulation. A study from Japan found that 14 days of gluten-rich diet might not increase serum levels of IgA ICs in IgAN patients.^[12]^ The relationship between GFD and IgAN still needs further probing.

Mendelian randomization (MR) is a method of inferring the causal effect of exposure on outcomes using genetic variants. Genetic variants are randomly assigned and independently located at the time of conception.^[13]^ With the publication of a large amount of genome-wide association study (GWAS) data and the advent of the era of precision medicine,^[14]^ MR study can effectively circumvent the limitations posed by insufficient sample size, high cost, ethical dilemmas, confounding factors, and reverse causality in conventional epidemiological studies. This article adopts the 2-sample MR analysis to explore the causal link between GFD and IgAN, which provides new perspectives for the prevention and treatment of IgAN.

## 2. Materials and methods

### 2.1. Study design

In this study, MR analysis was performed utilizing data published by GWAS summary statistics. GFD was used as the exposure, single nucleotide polymorphisms (SNPs) strongly associated with GFD were selected as instrumental variables (IVs), and the outcome variable was IgAN. Statistical analysis was conducted using the TwosampleMR package in R 4.3.1 software (R Foundation for Statistical Computing, Vienna, Austria) to evaluate the causal association between GFD and IgAN.

**Figure 1.**
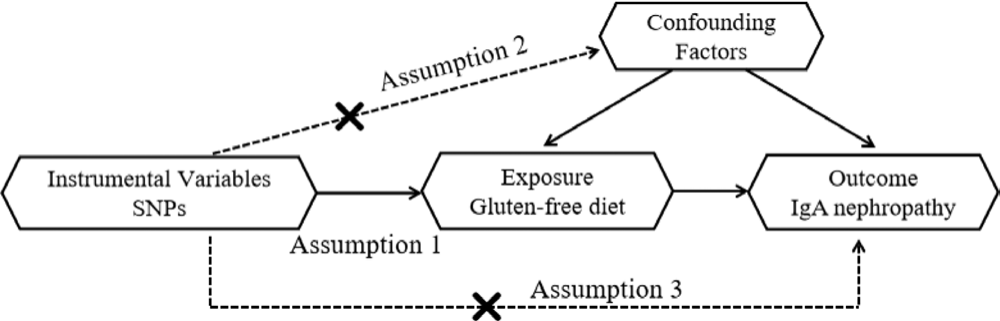
Schematic diagram of the MR analysis. MR = Mendelian randomization, SNP = single nucleotide polymorphism.

Whether genetic variants are valid IVs is a pivotal factor in assessing whether exposure has a causal effect on outcome in MR studies.^[15]^ Three core assumptions need to be fulfilled for selected genetic variants to be used as IVs in MR studies: assumption 1 that genetic variants are strongly associated with the exposure (correlation assumption); assumption 2 that genetic variants do not correlate with the outcome through confounders (independence assumption); and assumption 3 that there is no other pathway for genetic variants to be related to the outcome other than by exposure (exclusivity assumption).^[16]^ The schematic diagram of the MR analysis is presented in Figure 1.

### 2.2. Data source

Data for this study were obtained from the openGWAS database developed by the Medical Research Council Integrative Epidemiology Unit at the University of Bristol (https://gwas.mrcieu.ac.uk). GFD-related data (GWAS ID: ukb-b-11189) originated from the publicly available data from the GWAS study by Elsworth et al in 2020,^[17]^ with a total sample size of 64,949, which included 1376 GFD cases and 63,573 controls, and a total number of 9851,867 SNPs. GFD in this data was defined as following a GFD or wheat-free diet daily. IgAN-related data (GWAS ID: ebi-a-GCST90018866) originated from the analysis of the GWAS study published by Sakaue et al in the journal *Nature Genetics* in 2021 with a total sample size of 477,784, which included 15,587 IgAN patients and 462,197 controls, and the total number of SNPs was 24,182,646.^[18]^ IgAN in this data was defined as a chronic glomerulonephritis with predominantly IgA deposition in the mesangial area, often accompanied by complement C3 and IgG deposition. The aforementioned data were from European populations, and the sexes are male and female.

### 2.3. Selection of IVs

SNPs significantly associated with GFD were extracted from GWAS data using *P* < 5 × 10^−6^ and minor allele frequency > .01 as filtering conditions, and in order to eliminate linkage disequilibrium resulting from close locus location, parameters (*r*^2^ < .001, windows = 10,000 kb) were set for screening. The *F* statistic was calculated for each SNP, *F *= [(N − *K* − 1)/*K*] × [*R*^2^/(1 − *R*^2^)], where N is the sample size and *K* is the number of IVs. *R*^2^ represented the proportion of exposure variance explained by genetic variants, and *R*^2^ was calculated using the “get_r_from_bsen” function. SNPs with *F* statistic >10 were filtered to avoid bias from weak IVs and to cater the correlation assumption that SNPs are strongly associated with exposure.^[19]^ Queries were carried out through the PhenoScanner database (https://ldlink.nih.gov) to ensure that the selected IVs were independent of currently known confounders, satisfying the independence assumption that SNPs were independent of confounding factors. The *P* values for SNPs outcome were all less than those for exposure, and direction determination was performed using Steiger filtering (*P* < .05) to assure the exclusivity assumption that SNPs were not related to the outcome.^[20]^

### 2.4. Statistical analysis

Multiplicative random-effects inverse variance weighted (MRE-IVW), MR-Egger regression, weighted median, weighted mode, simple median, maximum likelihood ratio, and penalized weighted median were used as analytical models for MR analysis. Inverse variance weighted is a meta-analysis of the Wald ratio values (estimating the causal effect of exposure on outcome with individual IVs, *β*_outcome_/*β*_*exposure*_) of selected IVs in MR analysis to obtain the overall causal effect.^[21]^ We used MRE-IVW as the primary analysis method, which is the most efficacious and commonly used analysis method in 2-sample MR studies.^[22]^ Even in the presence of heterogeneity in testing, the random-effects model can degrade the precision of estimates and widen confidence intervals (CIs) to support the causal effect of exposure on outcome, while other results remain unchanged. Sensitivity analysis was performed with the Cochran *Q* test, MR-Pleiotropy RESidual Sum and Outlier (MR-PRESSO) test, MR-Egger intercept test, and leave-one-out method to assess the robustness and reliability of the results. Cochran *Q* test was used for evaluating the heterogeneity among IVs, when *P* > .05 suggests that there was no heterogeneity, and vice versa, the random-effects model should be used. The global test in MR-PRESSO was used to detect whether there was widespread horizontal pleiotropy in selected IVs overall; the outlier test was used to exclude SNPs that deviated from the fitted line, reducing the impact of pleiotropy on causality; and the distortion test was used to appraise whether there was a major discrepancy in causal inference before and after the removal of outliers.^[23]^ MR-PRESSO reperformed MR analysis after eliminating outliers if any were found. MR-Egger does not constrain the regression line to pass through the origin. During regression, the existence of the intercept item is taken into account, allowing for the presence of genetic pleiotropy in IVs. If we assume that IVs do not possess pleiotropy, the intercept should be 0. The MR-Egger intercept item was statistically tested against 0 as a test statistic. When there was no statistical difference (*P* > .05), it was deemed that there was no horizontal pleiotropy.^[24]^ Conversely, assumption 3 was violated and the inverse variance weighted results were unreliable. The leave-one-out method was used to observe whether the general effect was biased after removing SNPs one by one, and if abnormal values were found, MR analysis would be performed again after removing those values.

## 3. Result

### 3.1. Instrumental variables

The screening was carried out based on IVs selection criteria. The “harmonise_data” function was used to perform allelic synergy of exposure data with outcome data. Palindromic sequences and incompatible SNPs were excluded, and finally, 4 SNPs were included as IVs. The basic information on IVs is summarized in Table 1.

**Table 1:** Basic information of SNPs strongly associated with GFD.

SNPs	EA	OA	EAF	*β*	SE	*P*	*F*
rs1548306	T	A	0.66627	−0.00727	0.00085	1.10 × 10^−17^	43.49
rs9271842	A	C	0.39116	0.00582	0.00088	4.30 × 10^−17^	73.34
rs9273595	G	C	0.26159	0.01016	0.00092	2.10 × 10^−28^	122.20
rs9277568	C	T	0.30563	0.00468	0.00086	6.30 × 10^−8^	29.26

EA = effect allele, EAF = effect allele frequency, F = F statistic, GFD = gluten-free diet, OA = other allele, SE = standard error, SNP = single nucleotide polymorphism.

### 3.2. MR analysis

**Figure 2.**
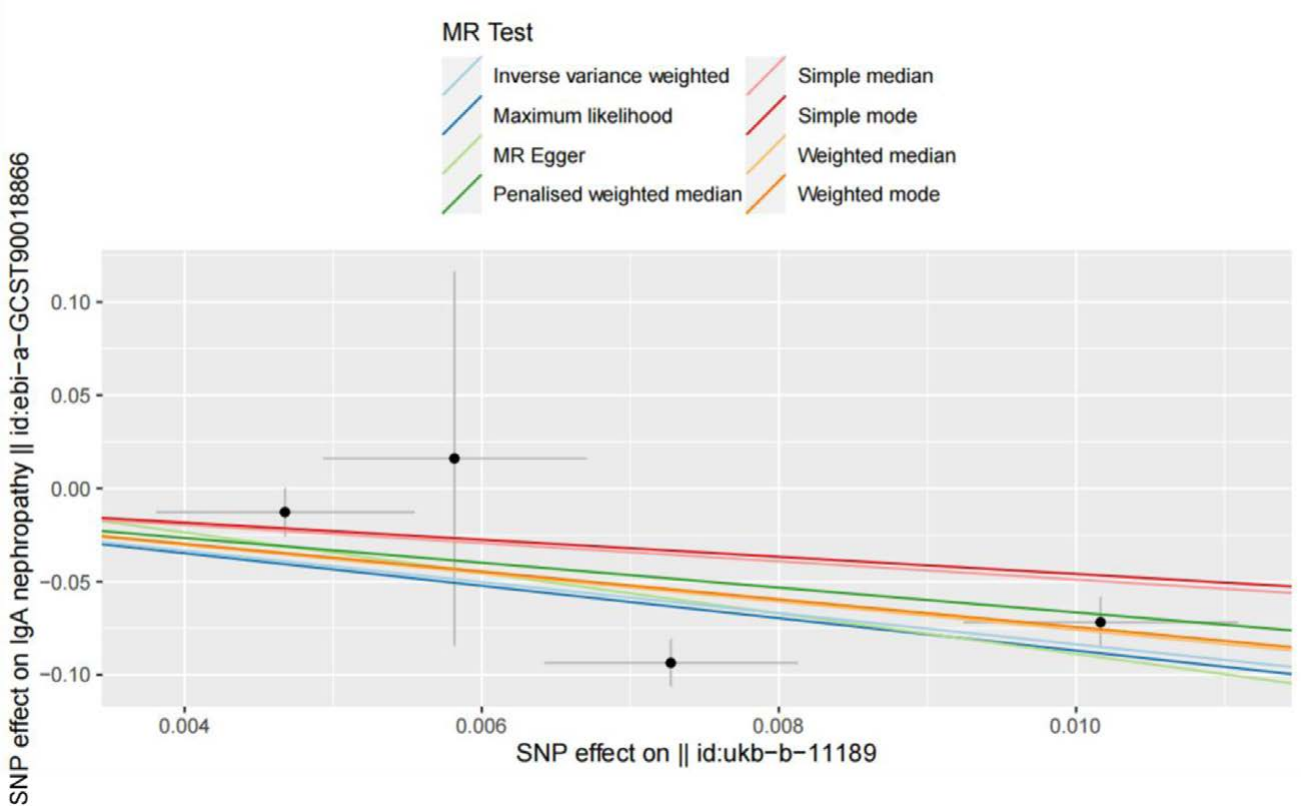
Scatter plot of the MR analysis. MR = Mendelian randomization, SNP = single nucleotide polymorphism.

Considering that the outcome variable was dichotomous, the odds ratio (OR) was chosen in this study to reflect the causal effect estimates. MRE-IVW model analysis displayed a significant negative association between GFD and IgAN (OR = 2.333 × 10^−4^, 95% CI = 4.628 × 10^−6^~1.176 × 10^−2^, *P *= 2.899 × 10^−5^), and its statistical power was calculated to be 100% using https://shiny.cnsgenomics.com/mRnd/. The results of MR-Egger regression, weighted median, weighted mode, simple median, maximum likelihood ratio, and penalized weighted median analytical models all showed that the OR values were <1, and the direction of the causal effect was consistent for 7 analytical methods. The scatter plot of the MR analysis is illustrated in Figure 2. The MR analysis results are listed in Table 2.

**Table 2:** MR analysis results.

Method	OR		95% CI	*P*
MRE-IVW		2.333 × 10^−4^	4.628 × 10^−6^~1.176 × 10^−2^	2.899 × 10^−5^
MR-Egger regression		1.913 × 10^−5^	1.772 × 10^−12^~206.518	0.319
Weighted median		5.083 × 10^−4^	3.375 × 10^−5^~7.654 × 10^−3^	4.221 × 10^−8^
Weighted mode		5.895 × 10^−4^	2.831 × 10^−5^~1.228 × 10^−2^	1.720 × 10^−2^
Simple median		7.521 × 10^−3^	5.735 × 10^−5^~0.986	4.935 × 10^−2^
Maximum likelihood ratio		1.664 × 10^−4^	1.744 × 10^−5^~1.588 × 10^−3^	4.035 × 10^−14^
Penalised weighted median		1.295 × 10^−3^	8.545 × 10^−5^~1.961 × 10^−2^	1.625 × 10^−6^

CI = confidence interval, MR = Mendelian randomization, MRE-IVW = multiplicative random-effects inverse variance weighted, OR = odds ratio.

### 3.3. Sensitivity analysis

**Figure 3.**
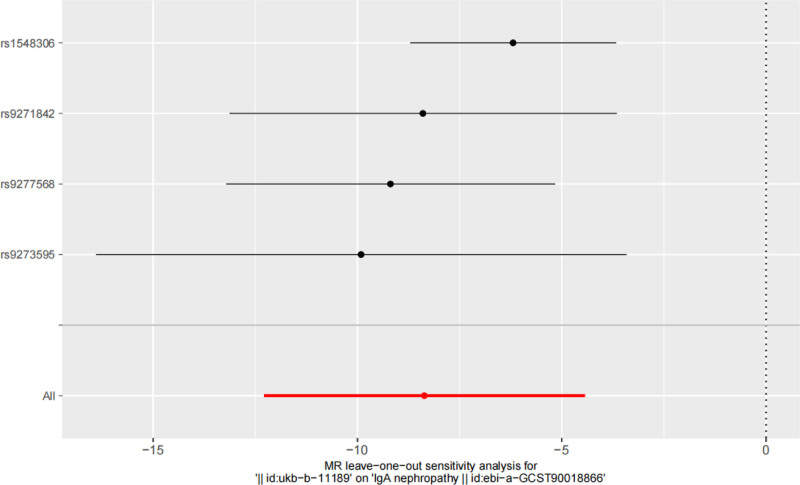
Results of “leave-one-out” analysis. MR = Mendelian randomization.

The heterogeneity test was conducted using the “mr_heterogeneity” function, where the *P* value of the Cochran *Q* test was .005 (*P* < .05) indicating that heterogeneity existed. The *P* value of Q_diff obtained using the “mr_rucker” function was .437 (*P* > .05), which denoted that the MRE-IVW model was more appropriate as the causal inference model for this study. The *P* value of the MR-PRESSO global test was .209 (*P* > .05), and no outliers were found. The *P* value of the MR-Egger intercept was .782 (*P* > .05) indicating that there was no horizontal pleiotropy in the selected SNPs. We used the leave-one-out method for visualization, excluding SNPs one by one. The CIs of the remaining SNPs were all to the left of 0, and no individual SNP was found to have a large impact on the results. The results of the “leave-one-out” analysis are depicted in Figure 3.

## 4. Current status of research on GFD and IgAN

GFD has been extensively proven as the only effective treatment for CD, and a large number of previous studies have demonstrated the protective effect of GFD on IgAN. A longitudinal study from Coppo’s team found that serum IgA ICs levels were significantly reduced in patients with primary IgAN who received 10 days of GFD, and that 10 days of diet containing no eggs or beef did not affect their serum.^[25]^ Serum IgA ICs were reduced to normal levels with 3 months’ GFD intervention,^[26]^ and in another descriptive study, the team found that primary IgAN patients had a notable decrease in circulating IgA ICs and improvement in both proteinuria and hematuria after 6 months of GFD.^[27]^ A study by Fornasieri et al showed that in 2 patients with primary IgAN whose hematuria had persisted for 29 months and 36 months, respectively, after 3 months and 5 months of GFD, the urinary red blood cells returned to the normal range, and IgA–antigliadin antibodies (IgA-AGAs) disappeared.^[28]^ Nagy et al determined serum antibodies against gluten, glycoprotein, α-lactalbumin, β-lactoglobulin, casein, and ovalbumin by enzyme-linked immunosorbent assay and noticed that compared with the normal group, the serum antibody titers in patients with IgAN increased significantly, indicating that the dietary antigen antibodies in some patients might be directly involved in the pathogenesis of IgAN.^[29,30]^ Laurent et al^[31]^ found that 70% of IgAN patients have high levels of IgA-AGAs in their serum and that gliadin could be a principal antigen involved in the formation of IgA ICs. Ovalbumin, bovine gamma globulin, and horse spleen ferritin were used as antigens for oral immunization of induced BALB/c mice for 14 weeks, and marked elevation of IgA was found in serum and excretory fluid, revealing that IgAN increased intestinal permeability.^[32]^ Follow-up of 27 primary IgAN patients following GFD for 1 year showed that IgA-AGAs disappeared in 80% of the patients, and the serum concentrations of IgA antibodies against ovalbumin and bovine serum albumin also decreased simultaneously, suggesting that gluten-containing diet possibly increases the permeability of the intestinal mucosa to other dietary antigens.^[33,34]^ Considerable proliferation of IgA-AGAs was observed in circulation and mesangium of BALB/c mice with a standard gluten diet or drinking water containing gliadin compared with ovalbumin-induced mice.^[35]^ A study from *The New England Journal of Medicine* indicated the diagnostic value of IgA-AGAs in differentiating IgAN from other primary glomerular diseases, with the sensitivity of 53%, specificity of 96%, positive predictive value of 89%, and negative predictive value of 77%.^[36]^ Clinical studies have discovered that IgA-AGAs correlate with age, disease course, blood pressure, and the progression of IgAN.^[37]^ In a patient with IgAN presenting with nephrotic syndrome and declining renal function, after 6 months of GFD, the patient’s serum creatinine and proteinuria decreased, while creatinine clearance and serum albumin increased.^[38]^ As a component of immune complexes, gliadin can promote the deposition of immune complexes in the mesangial area through its affinity for the mesangial area, and acting as a lectin can facilitate IgA and other molecules to bind to mesangial cells. In addition, gliadin combined with mesangial cells can also regulate arachidonic acid metabolism in the mesangium to stimulate the inflammatory response to play a pathogenic role.^[39,40]^ According to a case report from the Careggi University Hospital in Florence, after 2 years of GFD given to a young woman with CD and IgAN, thyroiditis, recurrent miscarriage, hyperlipidemia, and hyperamylasemia, renal function improved, proteinuria declined obviously, immunoglobulin A, lipase, and amylase in serum fell to the normal range, anti-β2-glicoprotein-1, antithyroglobulin, lupus anticoagulant, antigliadin, antiendomysial, and antitransglutaminase antibodies disappeared, and coagulation function restored to normal.^[41]^ In a 46-year-old male patient with focal proliferative IgAN and CD, hematuria and proteinuria subsided, and plasma albumin and hemoglobin returned to normal values after 8 months’ GFD in the absence of response to steroids combined with immunosuppressive agents.^[42]^ It has been indicated that approximately one-third of IgAN patients have gluten sensitivity in their intestinal mucosa.^[43]^ Renal deposition of tissue transglutaminase 2 (tTG2) was observed in some IgAN patients not undergoing GFD.^[44,45]^ Basic research from *Kidney International* explored the effect of a gluten diet on IgAN patients using transgenic IgAN mouse models expressing both human IgA1 and CD89 (α1KI-CD89Tg mice). The results demonstrated that after GFD, the complex of IgA1 and soluble CD89 (IgA1-sCD89) decreased in serum and mesangium, the level of IgA1-AGA reduced markedly in serum, the expression of transferrin receptor 1 (Tfr1) and tTG2 was alleviated in the mesangium, and renal IgA1 deposition and hematuria declined substantially. Levels of IgA1-AGA are associated with proteinuria, and long-term GFD is more effective than short-term intervention in IgAN progression. Studies suggested that the interaction of gliadin and CD89 aggravates IgAN through IgA1-sCD89 complex formation and mucosal immune responses probably. Early initiation of GFD might be a useful strategy for preventing the progression of IgAN.^[46,47]^ IgA-sCD89 complexes bound to mesangial cells via TfR1, inducing mesangial cell activation and overexpression of tTG2, and tTG2-TfR1 interaction exacerbated IgA1 deposition in the mesangium. Gluten diet led to increased tTG2 activity in the gut of humanized α1KI-CD89Tg mice, decreased TfR1 expression, IgA1-sCD89 and renal IgA deposition, and reduced hematuria in tTG2 knockout mice.^[48]^ A 24-year-old male patient with class I IgAN and CD presented with complete remission of nephropathy and improvement of clinical symptoms and laboratory test results of CD after 6 months of adhering to GFD under the general treatment model with angiotensin-converting enzyme inhibitors and oral iron supplements.^[49]^ Serum creatinine and urine protein creatinine ratio were normal in a 16-year-old boy with IgAN and CD after 5 years of following GFD.^[50]^

## 5. Discussion and conclusion

The clinical progression of IgAN is highly variable, with a diverse prognosis, posing tremendous challenges for its treatment. A retrospective cohort analysis from the UK National Registry of Rare Kidney Diseases showed that 50% of IgAN patients suffered renal failure or death during a median follow-up of 5.9 years, with the median renal survival time of 11.4 years, in which the mean age at renal failure or death was 48 years, virtually all patients being at risk of progressing to renal failure during their life expectancy, even patients who were conventionally at low risk had a high prevalence of developing renal failure within 10 years.^[51]^ IgAN patients have abnormal glycosylation of IgA1 at the O-terminal end of the hinge region in their serum, forming galactose-deficient IgA1 (Gd-IgA1). The formation of immune complexes containing abnormally glycosylated IgA1 is critical for the occurrence of IgAN. The precise pathogenesis of IgAN has not yet been elucidated, and the “four-hit” theory is currently recognized internationally: the presence of a large amount of Gd-IgA1 in the circulation; the triggering of an autoimmune response by Gd-IgA1, leading to the production of anti-Gd-IgA1 autoantibodies; autoantibodies bind to Gd-IgA1 to form pathogenic immune complexes; the immune complexes induce complement activation and inflammation when they penetrate the glomerular endothelial cell fenestrae.^[52]^ Gd-IgA1 is regarded as originating from gut-associated lymphoid tissue.^[53]^ Nefecon, which targets the release of budesonide to mucosal B cells (abundant in the Peyer patches-rich distal ileum) to reduce Gd–IgA1 production, has received widespread attention due to its high safety and tolerability in clinical trials. The introduction of Nefecon, the first oral targeted delayed-release formulation of budesonide for the allopathic treatment of IgAN, reaffirms the importance of gut mucosal immunomodulation in the management of IgAN.^[54]^ The gut–kidney axis mechanism remains instructive in future IgAN studies.^[53]^

For the first time, the potential causal relationship between GFD and IgAN was explored from the genetic perspective, with alleles following the principle of random assignment.^[55]^ The use of SNPs as IVs averted the analysis from being affected by confounders and reverse causality in traditional epidemiologic studies. The results of multiple analytical models were in convergence of direction, and sensitivity analysis simultaneously ensured the robustness of the results. Samples included in this study were European populations, and although population stratification bias was avoided, other populations need to be cautious in interpreting this result. This study was based on the GWAS database and failed to stratify the analysis according to other risk factors such as sex, age, and disease staging. In summary, this article analyzed the underlying causal effect between GFD and IgAN by MR approach and suggested that there might be a negative causal association between GFD and IgAN, advocating that GFD could be a promising remedy for treating IgAN, and that correcting nutritional deficiencies and achieving a well-balanced diet are also essential to the implementation of GFD.^[56]^

## Acknowledgments

We are thankful to Integrative Epidemiology Unit OpenGWAS project for providing publicly available summary data. We would like to appreciate the participants and researchers of GWAS Catalog and UK Biobank.

## Author contributions

**Conceptualization:** Tianqi Pei, Dengzhou Guo.

**Data curation:** Tianqi Pei.

**Formal analysis:** Tianqi Pei.

**Investigation:** Tianqi Pei.

**Methodology:** Tianqi Pei.

**Project administration:** Tianqi Pei.

**Resources:** Tianqi Pei.

**Software:** Tianqi Pei.

**Visualization:** Tianqi Pei.

**Writing – original draft:** Tianqi Pei, Dengzhou Guo.

**Funding acquisition:** Tianqi Pei, Dengzhou Guo.

**Supervision:** Dengzhou Guo.

**Validation:** Dengzhou Guo.

**Writing – review & editing:** Dengzhou Guo.
